# Production of HSVd- and PPV-free apricot cultivars by in vitro thermotherapy followed by meristem culture

**DOI:** 10.1186/s13007-025-01344-1

**Published:** 2025-02-20

**Authors:** C. Pérez-Caselles, L. Burgos, E. Yelo, L. Faize, N. Alburquerque

**Affiliations:** Group of Fruit Tree Biotechnology, Department of Plant Breeding, CEBAS-CSIC, Campus de Espinardo, Edif. 25, Murcia, 30100 Spain

**Keywords:** Etiolation, Heat treatment, *Hop stunt viroid*, *Plum pox virus*, *Prunus*

## Abstract

**Background:**

The production of virus-free apricots (*Prunus armeniaca* L.) is essential for controlling viral diseases, exchanging breeding materials without the risk of spreading new diseases, and preserving plant germplasm. *Plum pox virus* (PPV) is the most devastating disease of the *Prunus* genus and *Hop stunt viroid* (HSVd) is prevalent in most apricot-growing regions. It was evaluated whether thermotherapy, etiolation, or a combination of both followed by meristem culture could effectively eliminate PPV and HSVd from ‘Canino’ and ‘Mirlo Rojo’ apricot cultivars in vitro.

**Results:**

In the thermotherapy treatments, shoots were exposed to 38ºC and 32ºC, alternating every four hours, for 30, 35, 40, and 45 days. Before this, shoots were acclimated to heat for one day at 28ºC and two days at 30ºC. Etiolation experiments consisted of eight weeks of culture in dark conditions. A combination of 45 days of thermotherapy, as described previously, and etiolation was also performed. At the end of each treatment, 1.5 mm meristems were cultured, and developed as potential independent pathogen-free lines. The presence or absence of pathogens was analysed by RT-PCR. The 45 days of thermotherapy and the combined thermotherapy and etiolation treatments resulted in the highest percentages of PPV-free plants (66.7 and 75.0%, respectively). At least 40 days of thermotherapy were required to obtain HSVd-free plants, although the best efficiency was achieved at 45 days (22.7%).

**Conclusions:**

In this study, we have developed an effective in vitro thermotherapy protocol that eliminates PPV and HSVd from apricot cultivars. This is the first report where a thermotherapy protocol eliminates HSVd in *Prunus* species.

**Supplementary Information:**

The online version contains supplementary material available at 10.1186/s13007-025-01344-1.

## Introduction

Apricots (*Prunus armeniaca* L.) are one of Spain’s most economically significant fruit species, especially in the Region of Murcia, where edaphology and climate conditions ensure consistent and high-quality production. However, the presence of viral and viroid diseases can seriously compromise the productivity and quality of apricot trees. Rubio et al. [1] described 17 viruses and 2 viroids as pathogens that may infect apricot trees. *Plum pox virus* (PPV) is responsible for Sharka, the most serious disease affecting *Prunus* [2]. European and Mediterranean Plant Protection Organization (EPPO) guidelines for member countries consider PPV as a quarantine pest. Also, according to Spanish regulation (BOE-A-1995-14422), certified apricot trees must be free of the following viruses: PPV, and also *Apple chlorotic leafspot virus* (ACLSV), *Apple mosaic virus* (ApMV), *Apricot latent virus* (ApLV), *Prune dwarf virus* (PDV), and *Prunus necrotic ringspot virus* (PNRSV). Another highly significant pathogen is *Hop stunt viroid* (HSVd), as it is widespread and infects most of the crops in the area [3, 4]. All these results highlight the need for efficient methodologies to produce virus- and viroid-free *Prunus* trees for new plantations after removing infected trees from traditional plantings. Production of virus-free plants is necessary to control virus diseases, exchange breeding materials avoiding the danger of spreading a new disease, and preserve plant germplasm [5].

Plant viruses have been most efficiently eradicated by using in vitro plants which normally are maintained free from fungal and bacterial contamination and are available all year. The technique used traditionally for virus eradication is the meristem rescue (or shoot tip culture) which normally consists of isolating the apical dome and a few leaf primordia from in vitro shoots. Meristem culture is often insufficient to produce virus- and viroid-free plants by itself, because pathogens can rapidly infect meristematic cells sometimes, requiring the use of additional techniques [6].

In developmental biology, photomorphogenesis is light-mediated development, where plant growth patterns respond to the light spectrum. It is therefore several processes that affect development and aspects of the plant mediated by light [7]. Controlling these processes are photoreceptors, pigments that detect a range of the light spectrum and transfer the information to other cell components that translate the signals. The photoreceptors for red and far-red wavelengths are known as phytochromes and can have two different forms in the cell (Pr and Pfr) changing from one to other as a function of the red-light type. When plants are exposed to red light directly the Pr (inactive form) changes to Pfr (active form). In dark or shade conditions far-red wavelength predominates and the Prf form slowly changes back to the Pr form [8].

**Fig. 1:**
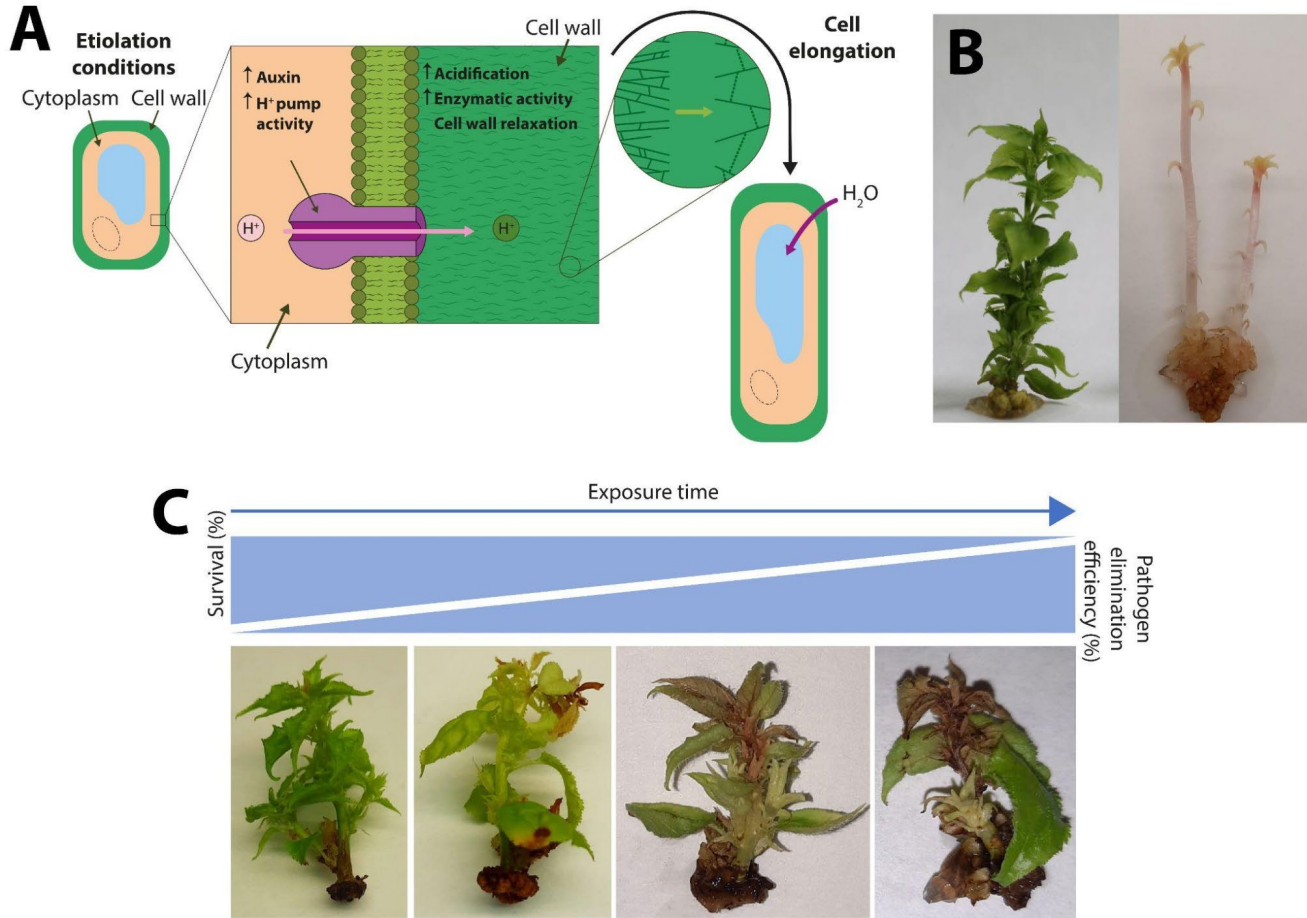
**A**) Physiological changes produced in the cells as a consequence of culturing the plants in darkness. **B**) Micropropagated ‘Ca-PPV’ shoots grown 4 weeks with a photoperiod versus 8 weeks in the dark (etiolated). **C**) Effect of high temperature exposure on micropropagated ’Ca-PPV’ shoots 30, 35, 40 and 45 days. Survival decreases with exposure time whereas probability of finding pathogen-free plants should increase

Among the different processes that phytochromes control is etiolation [9]. Etiolation is the process that happens to plants when they are in partial or total darkness which produces stem elongation, longer internodes and smaller leaves. Additionally, aerials parts are generally white or yellow (Fig. 1B). Colour is due to a total or almost total reduction of photosynthetic activity and consequent inhibition of chlorophyll. Searching for the source of light, plants elongate stems by triggering the production of auxins in the stem apex which is unidirectionally transported towards roots, inhibiting development of lateral buds and other organs such as leaves [10]. Auxins activate proton pumps within the plasmatic membrane producing acidification of the external medium. Low pH activates enzymes that loosen cell walls allowing cell growth by turgor pressure (Fig. 1A).

One of the most common methods used for virus eradication is thermotherapy followed by a meristem rescue. Thermotherapy involves growing and maintaining plants at moderately high temperatures. Treatment duration depends on the sensitivity of the viral pathogen and the physiological tolerance of the plant species [11]. Normally, a shoot tip culture is carried out at the end of the high-temperature exposure time. The thermotherapy regime selected should enable the plant to survive while effectively inactivating the virus, leading to the growth of virus-free shoot tips. Typically, extended heat treatment can enhance the likelihood of virus eradication, but it also decreases the viability of the treated explants (Fig. 1C), as host plants are often sensitive to such conditions.

The mechanism by which thermotherapy is effective in eliminating plant viruses is unknown. However, the main hypotheses [12] are: (1) Virus movement toward meristematic cells is inhibited by high-temperatures (35 to 42 °C), (2) reduced viral replication, (3) Degradation or silencing of RNA, decreasing the virus titter in infected shoot tips.

The application of thermotherapy followed by meristem rescue has been effective in eliminating several viruses in *Prunus* species, such as PPV in apricot [13], PNRSV in plum and peach [14, 15], and ACLSV in almond [16]. However, to the best of our knowledge, HSVd has not been successfully eliminated from *Prunus* species through thermotherapy. In our laboratory, we have the apricot varieties ‘Canino’ infected with PPV (Ca-PPV) and ‘Mirlo Rojo’ infected with HSVd (MR-HSVd). The objective of this work is to develop a thermotherapy protocol, etiolation, or a combination of both, followed by meristem rescue that allows the elimination of both pathogens from these apricot varieties.

## Methods

### Plant material

Micropropagated Ca-PPV and MR-HSVd were used for this study. Both cultivars are micropropagated in 500 mL glass jars, around 12 shoots per jar, in a semisolid medium for apricot shoots multiplication (SSM) described by Wang et al. [17]. The pH was adjusted to 5.7 before autoclaving at 121 °C for 20 min. Apricot plants were maintained in vitro by culturing to fresh medium every four weeks in a culture chamber at 23 ± 1 °C, 16/8 h light/darkness photoperiod (56 µmol m^− 2^ s^− 1^).

### Treatments for virus and viroid eradication

#### Thermotherapy experiments

Ca-PPV and MR-HSVd shoots (1.5 cm in length) containing the apical and axillary buds were cultured in a growth chamber (BINDER KBF 1020, BINDER Gmbh, Tuttlingen, Germany) using the standard conditions described above but changing temperature parameters. Experiments were initiated by culturing explants at 28°C one day and at 30°C the following two days to acclimatise shoots to the treatment. Afterwards, the temperature was alternated between 38°C and 32°C every four hours for 30, 35, 40, or 45 days.

#### Etiolation experiments

Ca-PPV and MR-HSVd shoots (around 15 mm in length) containing the apical and axillary buds were cultured using standard temperature conditions but in darkness, during eight weeks.

#### Combined experiments

The combined experiment consisted in exposing Ca-PPV and MR-HSVd shoots to 45 days of thermotherapy (as described in Sect. 2.1) in dark conditions.

### Shoot tip recovery and meristem growth

At the end of the exposure times of each experiment, healthy apical and axillary meristems of approximately 1.5 mm were isolated, avoiding primordia leaves (if possible), and cultured on Meristem Multiplication Medium 1 in 9 cm Petri dishes (MM1, Supplementary Fig. 1). Briefly, MM1 consisted of QL [18] macronutrients, DKW [19] micronutrients and vitamins, 2% (w/v) sorbitol, 0.56 µM BAP_R_, 0.05 µM IBA, 1.05 µM meta-topolin, 14.8 µM adenine, and agar 0.7%. During the first seven days in MM1, indirect light was used.

After 4 weeks, meristems were transferred to Meristem Multiplication Medium 2 (MM2) described by Pérez-Tornero et al. [20]. MM2 was based on QL macronutrients and micronutrients, vitamins described in Pérez-Tornero et al. [20], 2% (w/v) sorbitol, 6.6 µM BAP, 0.05 µM IBA, and agar 0.7%. Both meristem media pH was adjusted to 5.7 before autoclaving at 121 °C for 20 min. After 4 weeks, the explants were transferred to SSM medium until independent lines were established. An independent line was defined as a potential pathogen-free plant proceeding from one individual meristem. The time necessary from the rescue of a meristem to the establishment of a shoot was 12 weeks, and at this moment, the shoots have enough plant material for the first in vitro evaluation (pathogen presence/absence analysis by RT-PCR).

Those lines where the presence of the pathogen was detected were eliminated. Potentially pathogen-free lines were multiplied on SSM medium and subcultured every 4 weeks. After 12 weeks, 24 weeks from meristem rescue, the second in vitro evaluation was performed to verify results from the first evaluation.

### Rooting and acclimatisation

Potentially pathogen-free lines were rooted and acclimatised (Supplementary Fig. 1) as described by Pérez-Caselles et al. [21]. Six shoots per line of at least 15 mm long, growing in SSM, were rooted in the medium previously described by Pérez-Tornero & Burgos [22]. Almost all plants rooted and were placed in 300 mL pots within zip plastic bags in the greenhouse, which were gradually opened when new growth was observed. The *ex vitro* evaluation (the third evaluation) was carried out with these acclimatised plants.

### Pathogen detection

The presence or absence of the pathogens in the shoots grown from the meristems were analysed by RT-PCR. Leaf samples from each shoot (100 mg) were harvested and frozen in liquid nitrogen. Total RNA extraction was carried out using a NucleoSpin RNA Plant and Fungi kit (Macherey-Nagel, Düren, Germany). The quality and concentration of extracted RNA were measured using a NanoDrop. For cDNA synthesis, M-MLV retrotranscriptase (Promega, Madison (WI), USA) was used. PCR for the detection of PPV and HSVd (Table 1, Supplementary Fig. 2) in ‘Canino’ and ‘Mirlo Rojo’ shoots, respectively, was performed using GoTaq^®^ Green Master Mix (Promega). Lastly, PCR products were observed by an electrophoretic analysis using Green Safe (NZYTech, Lisboa, Portugal).

**Table 1:** Primers used for detection of PPV and HSVd in apricot cultivars

Primer	Sequence	Product size	Reference
PPV-F	5’-CAATAAAGCCATTGTTGGATC-3’	313 bp	[23]
PPV-R	5’- CTCTGTGTCCTCTTCTTGTG-3’		
HSVd-F	5’-AATTCTCGAGTTGCCGCAACA-3’	303 bp	[24]
HSVd-R	5’-CAGGGGCTCAAGAGAGGATC-3’		

Abbreviations: PPV, *Plum pox virus*; HSVd, *Hop stunt viroid*; bp, base pairs

### Statistical analyses

Survival and efficiency of virus and viroid-free plants production was analysed by Maximum Likelihood Anova. Examination of trends was carried out for the days that plants were exposed to the thermotherapy treatment. Specific contrasts were designed to compare the best duration of thermotherapy treatment with the dark and the combined experiments.

## Results

### Thermotherapy allowed efficient production of virus- and viroid-free plants

**Fig. 2:**
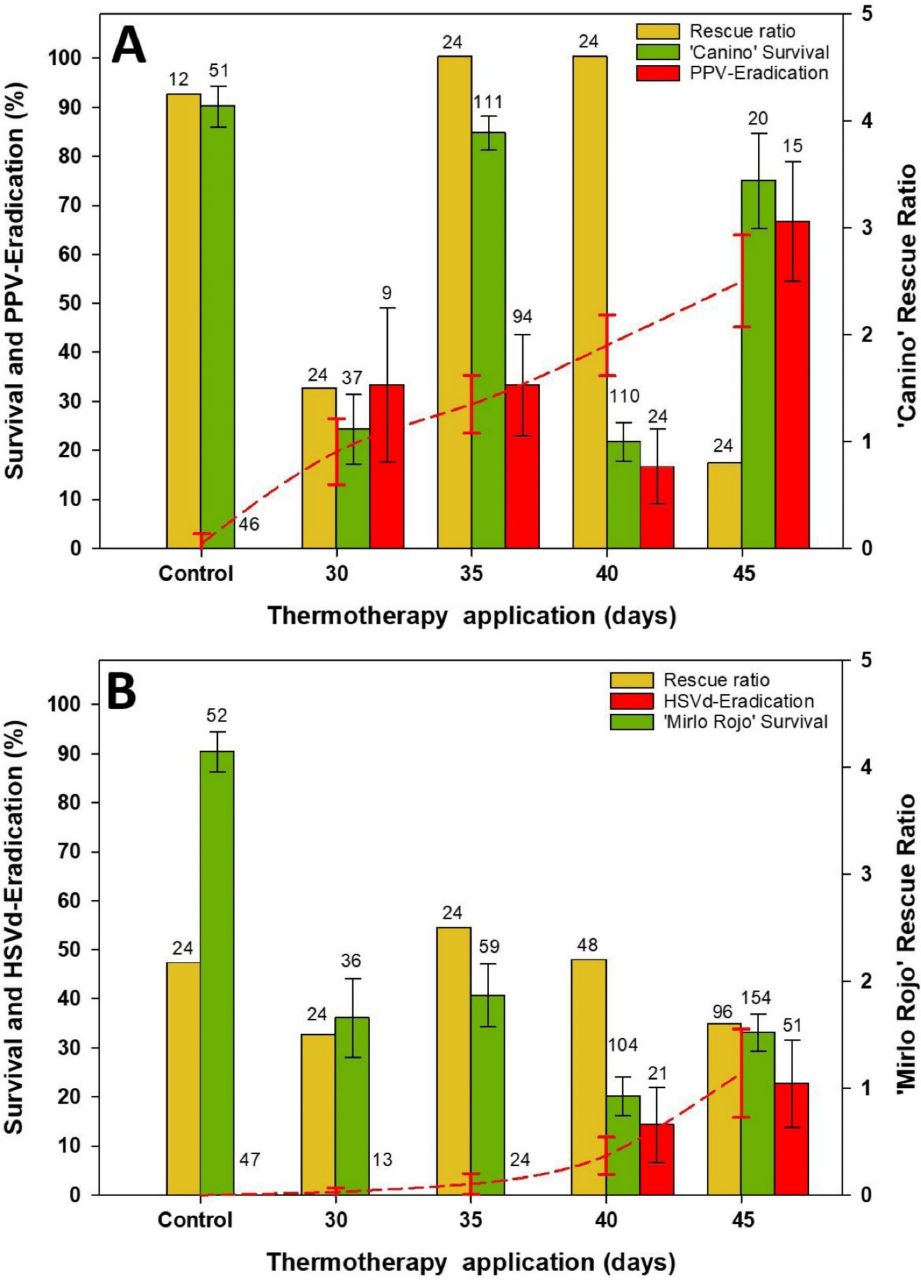
Effect of exposure time to thermotherapy treatment on rescue ratio, percentage of surviving meristems, and efficiency of PPV elimination (**A**) as well as HSVd elimination (**B**). Thermotherapy treatments consisted of cycles of 32–38ºC every four hours applied to Ca-PPV and MR-HSVd apricot shoots. Error bars represent the standard error. The dashed line represents the trend in pathogen eradication. The numbers indicate the initial treated shoots on yellow bars, the total number of isolated meristems on green bars, and the number of established lines on red bars

In vitro Ca-PPV and MR-HSVd microshoots were exposed to high temperatures for different periods of time. Rescue ratio of meristems from control microshoots was over 4.3 in Ca-PPV and more than 2.2 in MR-HSVd (Fig. 2A and B). This rate is calculated as the average number of meristems rescued at the end of heat treatment from the microshoots placed initially. Meristems of around 1.5 mm in length and without apparent necrosis were rescued. Interestingly, in both cultivars the rescue ratio decreased after 30 days (1.5 for both cultivars) of exposure to high temperature, but after 35 (4.6 for Ca-PPV and 2.5 for MR-HSVd) and 40 days (4.6 for Ca-PPV and 2.2 for MR-HSVd), the plants recovered and the number of meristems that could be isolated increased. Finally, after 45 days (0.8 for Ca-PPV and 1.6 for MR-HSVd) of thermotherapy treatment the rescue rate decreased in both cultivars (Fig. 2A and B).

Survival of rescued meristems was significantly affected by treatment duration in both cultivars (*P* < 0.001). There was a high survival of control (non-treated) meristems. After 30 days survival of treated meristems decreased abruptly, largely recovering after 35 days of treatment and decreasing again after 40 days. However, survival of rescued meristems increased again after the 45 days treatment indicating that the lowest rescue rate, found at this time, was of meristems that were in good conditions (Fig. 2A and B).

The efficiency of pathogen elimination (Fig. 2A and B) correspond to the ex-vitro evaluation. The percentage of Ca-PPV plants found virus-free after the thermotherapy treatments tended to significantly increase (*P* < 0.01) with the days of treatment. Nevertheless, virus-free plants were produced in all the treatments, ranging from 16.7 to 66.7% (Fig. 2A), being 45 days the most efficient treatment. A similar significant trend (*P* < 0.05) was found in MR-HSVd plants although only the longest treatments produced viroid-free plants in this case (Fig. 2B). Efficiencies of HSVd-free plants were lower than those of PPV-free plants and ranged from 14.3 to 22.7% after 40 and 45 days of treatments, respectively.

### The combination of thermotherapy and dark treatment did not improve the efficiency producing virus or viroid-free plants

**Fig. 3:**
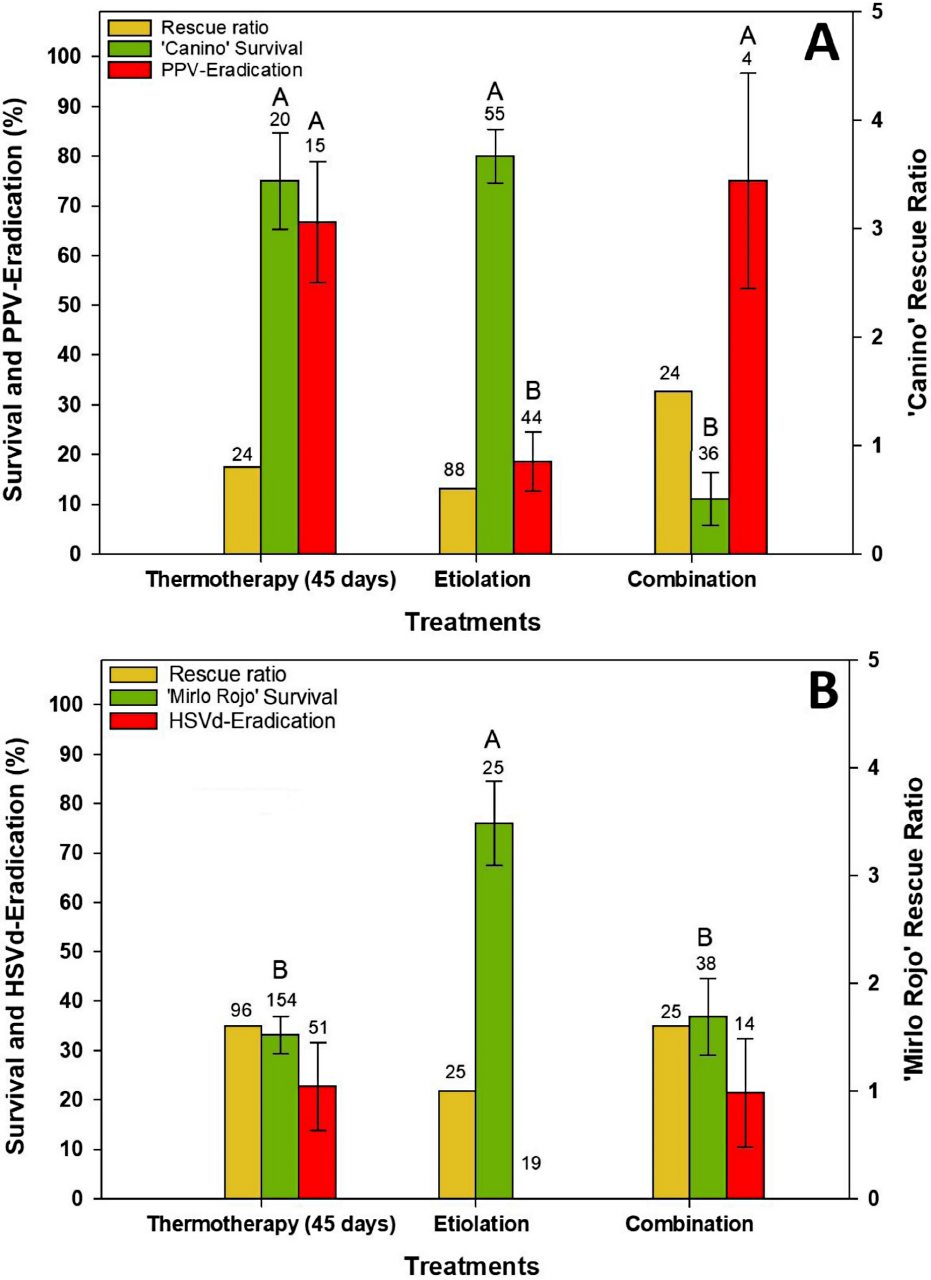
Effect of 45 days exposure to thermotherapy, etiolation, or combination of both treatments on meristem rescue ratio, percentage of surviving meristems, and efficiency of PPV elimination (**A**) as well as HSVd elimination (**B**). Error bars represent the standard error. The numbers indicate the initial treated shoots on yellow bars, the total number of isolated meristems on green bars, and the number of established lines on red bars. Different letters indicate significant differences (*P* < 0.05) between treatments, after specific contrast, for each evaluated parameter

Results of 45 days thermotherapy, etiolation and combination of these treatments are shown in Fig. 3. The rescue rate ranged from 0.6 to 1.5 in the case of Ca-PPV and from 1.0 to 1.6 in MR-HSVd, with the lowest value corresponding to the etiolation treatment in both cultivars.

Survival of Ca-PPV and MR-HSVd behaved quite differently depending on the treatment. Although in both cases there were significant differences between treatments (*P* < 0.01), in Ca-PPV these differences were due to a low survival in the combined treatment but in MR-HSVd they were due to a high survival of meristems after dark conditions (Fig. 3A and B).

Production of PPV-free ‘Canino’ plants was significantly affected by treatment (*P* < 0.01). 45 days thermotherapy was similar to the combined treatment, and both were significantly better than etiolation with efficiencies of 66.7% and 75.0%, respectively (Fig. 3A).

A similar situation was found in MR-HSVd treated plants, although the treatment did not have a significant effect. No viroid-free plants were found in the dark treatment. However, HSVd was eradicated in 45 days of thermotherapy and the combined treatment with efficiencies of 22.7% and 21.4%, respectively (Fig. 3B).

The efficiency values of the treatments depicted in Figs. 2 and 3 correspond to those obtained in the ex vitro evaluation. In the second in vitro evaluation, false negatives were found in the treatments of 30 and 35 days of thermotherapy in Ca-PPV (1 plant from each treatment) and 45 days of thermotherapy in MR-HSVd (2 plants). However, the ex vitro evaluation confirmed the data from the second in vitro evaluation, since no false negatives were found.

### Production of virus- and viroid-free plants was associated with the meristem but not the shoot where the meristem proceeded

After exposing in vitro shoots to the different treatments more than one meristem was frequently rescued from each plant. We decided to identify those meristems rescued from the same shoot from some treatments (MR-HSVd meristems from 40 to 45 days of thermotherapy and meristems of both cultivars from etiolation treatments). The objective was to determine whether the treatment affected all meristems isolated from the same shoot equally. Table 2 shows the RT-PCR evaluation of lines produced (A-D) from each of those specific shoots (1–4). Negative RT-PCR lines mean absence of the pathogen after two in vitro evaluations and one additional evaluation in the greenhouse.

**Table 2:** Comparison of RT-PCR evaluation between lines coming from the same treated shoot

Experiment	Cultivar	Shoot	Line	RT-PCR^a^
Thermotherapy 40 days	MR-HSVd	1	A	+
			B	-
			C	+
		2	A	+
			B	+
		3	A	+
			B	+
Thermotherapy 45 days	MR-HSVd	1	A	+
			B	-
		2	A	+
			B	+
		3	A	+
			B	+
			C	+
			D	+
		4	A	+
			B	-
Etiolation	Ca-PPV	1	A	+
			B	+
			C	-
		2	A	-
			B	+
	MR-HSVd	1	A	+
			B	+

^a^“ +” and “-” mean the presence and absence of the pathogen in the evaluated line, respectively

There were shoots that did not produce pathogen-free plants because all the rescued meristems remain infected. Additionally, shoots that produced only pathogen-free meristems were not found. Whenever a pathogen-free line was found, at least another line from the same shoot remained infected. These data point to the need to study and evaluate each line independently, regardless of the original shoot they proceed.

## Discussion

The factors to be defined in the development of a thermotherapy protocol are the temperature and the exposure time of the treatment, as they are limited by the tolerance of the host plant [25]. In general, the higher the exposure temperature, the greater the pathogen elimination (treatment efficiency) and the lower rate of plant survival. The success of heat treatments lies in finding a balance that maximises both parameters, survival and pathogen elimination (Fig. 1C). Effective in vitro thermotherapy protocols have been described for the elimination of ACLSV and PNRSV in myrobalan [25], PNRSV in plum [25], and PPV and PNRSV in peach [26].

The application of thermotherapy followed by meristem culture offers a high success rate in obtaining pathogen-free plants [13, 27]. Generally, the smaller the rescued meristem size, the higher the probability of pathogen eradication [12]. Nevertheless, meristem size is a crucial factor in their survival. Karimpour et al. [28] were unable to obtain any surviving apricot meristems when sizes were between 0.2 and 0.5 mm, while Zarghami & Ahmadi [29] reported a considerable increase in survival for 1 mm meristems compared to 0.5 mm and 0.2 mm meristems in peach. Choosing an appropriate thermotherapy regime can inhibit viral replication and reduce virus movement towards meristematic cells, enabling the excision of a larger meristem portion, thus increasing the probability of meristem regeneration [30]. The rescue of meristems between 1 and 2 mm after thermotherapy successfully produced ACLSV-, ApMV-, and *Tobacco ringspot virus* (TRSV)-free apricot [27, 31], *Cherry virus A* (CVA)-free plum [32], and PNRSV-, ACLSV-, and PPV-free peach [27, 29, 33], as well as PNRSV- and PDV-free cherry shoots [27].

*Prunus* species, especially apricot, are susceptible to prolonged exposure to high temperatures [15, 34]. Zarghami & Ahmadi [29] reported low and zero survival percentages of meristems when peaches were exposed to 39ºC for 10 and 15 days, respectively. It has been reported that maximum temperatures of 38ºC have allowed the survival of plum [14, 32], peach [15, 29], and almond meristems [16]. Prolonged exposure of apricot to 37ºC for 15 to 21 days achieved meristem survival between 80% and 100%, while survival decreased to 37.5% after 32 days of exposure [27]. Karimpour et al. [28] reported 0% survival in apricot meristems after exposing plants to maximum temperatures of 38ºC for 60 days. Cieslinska [25] recommended acclimatising *Prunus* species before thermotherapy by gradually increasing the temperature. This approach has been implemented in many protocols and was also used in this study.

Walkey & Freeman [35] observed that alternating cycles of high and low temperatures inactivated *Cucumber mosaic virus* (CMV) in infected *Nicotiana rustica* tissue cultures. Stein et al. [15] reported that alternating between 38ºC and 28ºC was more effective for eliminating PNRSV in peach than using a constant high temperature. Alternating high and low temperatures in thermotherapy protocols has been effective for eliminating PPV, PNRSV, and PDV in apricot [36], PNRSV and CVA in plum [14, 32], and ACLSV, ApMV, and *Tomato ringspot virus* (ToRSV) in almond [16]. Furthermore, Howell et al. [37] applied alternating cycles between 40ºC and 32ºC every 4 h in sweet cherry.

In this study, a thermotherapy protocol combined with meristem rescue was developed, successfully eliminating PPV and HSVd from the apricot cultivars ‘Canino’ and ‘Mirlo Rojo’. Figure 1C shows the deterioration suffered by apricot shoots with time exposed to thermotherapy treatment. Surprisingly, a higher rescue rate and survival was achieved after 35 days than after 30 days in both varieties. This can be explained by the fact that a shorter exposure period (30 days) may not be sufficient to induce an adaptive response in plant tissues. At the beginning of the heat treatment, cells experience stress, which may negatively affect the development and viability of the buds. Plants respond to heat stress through modifications in their development, physiology, and biochemistry, regulated by the expression of stress-responsive genes [38]. The complex signalling network that triggers the early response to high temperatures involves the action of Reactive Oxygen Species (ROS), calcium ion (Ca²⁺) flux, phospholipids, and phytohormones. The interaction of these components activates various classes of transcription factors, leading to a cascade of events that determines the expression of heat-responsive genes [39]. However, when the exposure time is increased to 35 days, it is possible that the plants have activated mechanisms of response to heat stress, allowing a greater number of buds to develop under optimal conditions. Plants constantly face challenges to survive under several environmental stress conditions, including high temperatures. In long-term response to high temperatures, plants modify their metabolic processes in various ways, especially by producing compatible solutes that help stabilise proteins and cellular structures, maintain cell turgor through osmotic adjustment, and enhance the antioxidant system to restore cellular redox balance and homeostasis [40]. After 40 days of treatment, the rescue rate remains stable, but survival decreases drastically. This suggests that prolonged heat exposure may have caused irreversible damage to the rescued meristems, compromising their further development.

For both Ca-PPV and MR-HSVd, the best treatment was 45 days. Although the rescue ratio in this treatment was low (few meristems were isolated after treatments), meristem survival was high, and the best pathogen elimination efficiency was achieved, with 66.7% for PPV and 22.7% for HSVd. Koubouris et al. [13] achieved 82% efficiency in PPV elimination in apricot, while Gella & Errea [27] reported an efficiency rate for ACLSV elimination between 66% and 100%. Other pathogens such as ApMV, TRSV, PDV, and PNRSV have also been eliminated from apricot [31, 36]. On the whole, the use of thermotherapy followed by meristem rescue is an effective technique for the eradication of viruses and we have demonstrated for the first time its feasibility for eliminating a viroid in apricot.

One method not widely used for virus elimination is stimulating stem elongation in shoots to expand the virus-free region, followed by meristem rescue. Chen et al. [41] observed an 82% increase in the length of apple shoots when they added 15 µM melatonin to the culture medium. After four weeks, meristems from the treated plants were isolated, achieving 85% of survival and 95% of plants *Apple stem grooving virus* (ASGV)-free. In our study, apricot shoots were etiolated, and meristem rescue was carried out when the stems were elongated. The rescue rate was less than 1 for both Ca-PPV and MR-HSVd, but survival was over 75% in both varieties. The PPV elimination efficiency was 18.6%, whereas it was not possible to find HSVd-free plants using this technique. Etiolation does not require specialised equipment and is simpler than thermotherapy, but it proves to be much less effective and not all pathogens were eliminated.

Since the etiolation experiment had no effect on HSVd elimination, the results of the combined experiment (thermotherapy and etiolation) were similar to those from thermotherapy. However, the combination treatment achieved a PPV elimination efficiency of 75%, improving upon the thermotherapy treatment alone, although it was not significantly better. The combination of techniques often results in greater pathogen elimination efficacy, especially for those that rapidly infect the meristematic region [42]. The combination of thermotherapy and chemotherapy successfully obtained PNRSV-free [25] and CVA-free plums [32], PDV-free cherry [25], and PNRSV- and ACLSV-free Myrobalan plum [25].

Not only apical meristems can be used in pathogen elimination protocols, but also axillary buds. Cheong et al. [43] found no significant differences between using apical or axillary meristems for the elimination of viruses affecting sugarcane. In the thermotherapy experiments, both apical and axillary buds that appeared healthy at the end of the exposure times were rescued. In the etiolation experiments, only apical meristems were rescued since dark conditions inhibit the production of lateral buds. However, the same shoot could produce the elongation of two or more independent stems (Fig. 1B), allowing the rescue of more than one meristem from the same initial shoot. To determine whether the pathogen-free line obtained was related to the treated shoot, traceability of meristems rescued from the same shoot was identified until they became lines and were evaluated (Table 2). In this study, it was demonstrated that each rescued meristem must be analysed independently, as several lines originating from the same treated shoot can produce different RT-PCR results. This could be due to the fact that treatments do not affect all buds on the plant equally, or to the erratic distribution of some viruses in the plant [44, 45].

The combination of thermotherapy with tissue culture is the most commonly used technique for pathogen elimination [11]. However, heat treatments are often ineffective for eradicating certain viroids. This could be due to their structural simplicity, which facilitates movement between cells, their simple and efficient replication mechanism, or the absence of protein structures susceptible to degradation by heat. El-Dougdoug et al. [46] reported the inefficiency of thermotherapy followed by meristem rescue in eliminating HSVd in peach and pear plants. An alternative for these pathogens is the use of cold therapy, which consists of exposing plants to low temperatures, followed by meristem rescue [47]. El Doug-doug et al. [46] achieved an 18% efficiency in HSVd elimination in peach by keeping the shoots at 4ºC for three weeks, followed by a meristem rescue.

Nevertheless, heat therapy has been reported as useful for eliminating certain viroids, such as *Apple scar skin viroid* (ASSVd) in pear [48] or *Chrysanthemum stunt viroid* (CSVd) [49]. Additionally, Matousek et al. [50] reported a reduction of 70–90% in *Hop latent viroid* (HLVd) viral particles when hop plants were exposed to 37ºC for two weeks. This indicates that viroid elimination is possible if the right protocol is found. To the best of our knowledge, this study is the first report of HSVd elimination using thermotherapy followed by meristem rescue. We have observed that the exposure time of the treatment is a determining factor, as at least 40 days were needed to find HSVd-free plants, although the best efficiency was obtained after 45 days.

## Conclusions

In this study, we have developed an effective in vitro thermotherapy protocol that eliminates PPV and HSVd from apricot cultivars. This is the first report where HSVd is eliminated from a *Prunus* species by using thermotherapy. The time of exposure has been a determining factor in HSVd elimination since long periods are necessary for success. Etiolation conditions allowed the production of PPV-free apricot plants with low efficiency, but HSVd could not be eradicated with this technique. Combination of thermotherapy and etiolation also allowed the eradication of both pathogens.

## Electronic supplementary material

Below is the link to the electronic supplementary material.

Supplementary Material 1

## Data Availability

No datasets were generated or analysed during the current study.

## Abbreviations

ACLSV: Apple chlorotic leafspot virus
ApMV: Apple mosaic virus
ASGV: Apple stem grooving virus
ASSVd: Apple scar skin viroid
Ca-PPV: ‘Canino’ infected with Plum pox virus
CMV: Cucumber mosaic virus
CSVd: Chrysanthemum stunt viroid
CVA: Cherry virus A
HLVd: Hop latent viroid
HSVd: Hop stunt viroid
MM1: Meristem medium 1
MM2: Meristem medium 2
MR-HSVd: ‘Mirlo Rojo’ infected with Hop stunt viroid
PDV: Prunus dwarf virus
PNRSV: Prunus necrotic ringspot virus
PPV: Plum pox virus
SSM: Shoot semisolid medium
ToRSV: Tomato ringspot virus
TRSV: Tobacco ringspot virus
