# Supplementary material for: Production of HSVd- and PPV-free apricot cultivars by in vitro thermotherapy followed by meristem culture
Source: Plant Methods. 2025 Feb 20;21:23. doi: 10.1186/s13007-025-01344-1 (PMC11843746; doi:10.1186/s13007-025-01344-1)
Supplement: Supplementary file 1 — Supplementary Material 1 [file 13007_2025_1344_MOESM1_ESM.docx]

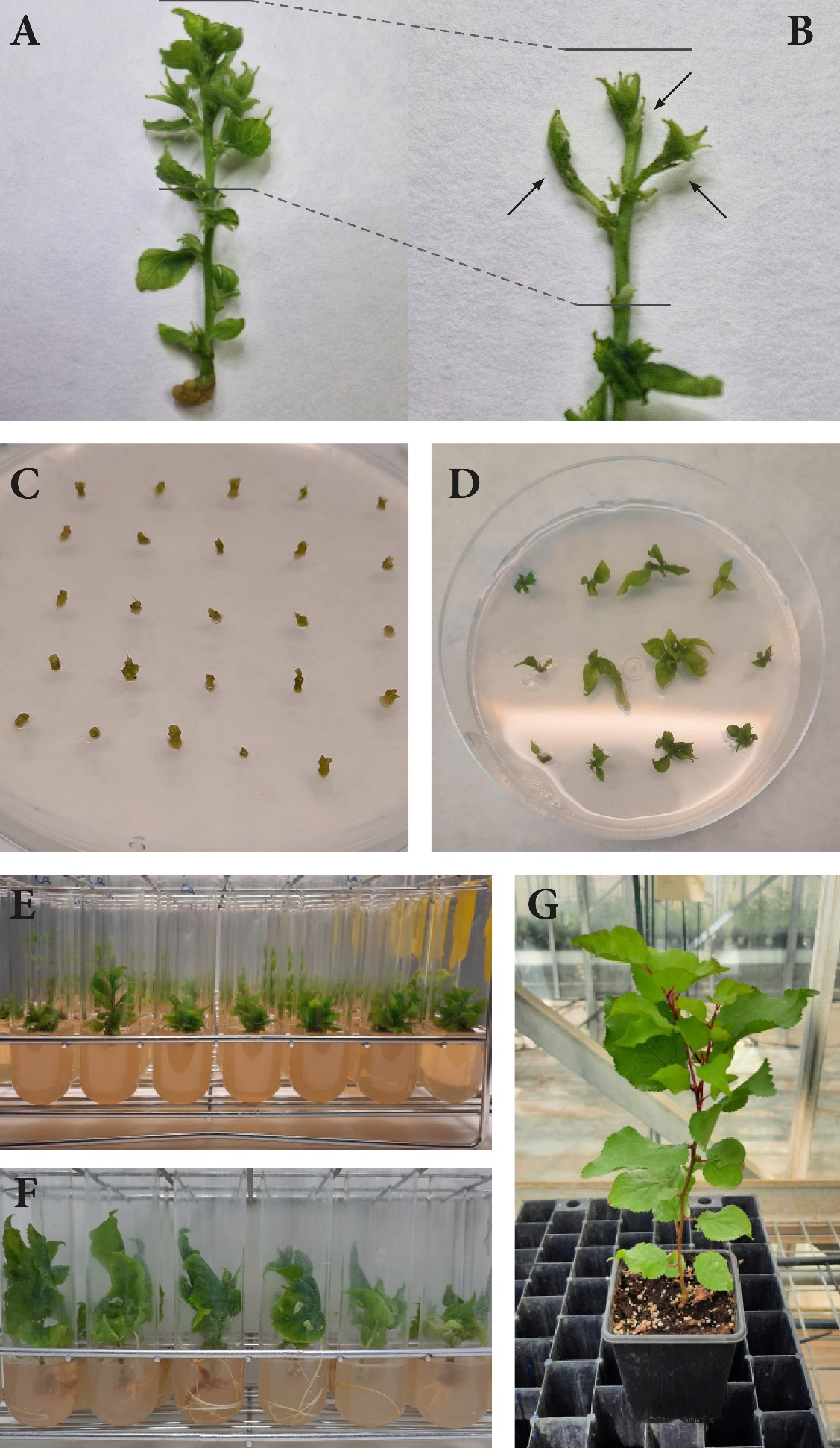


Supplementary figure 1. Apricot shoots (A) are used for the meristem rescue. Arrows indicate the type of meristem rescued (B). Once they were excised, primordia leaves were removed and meristematic dome cultured (C). Isolated meristems after 6 (D) and 12 (E) weeks. Rooted (F) and acclimatised (G) plants.


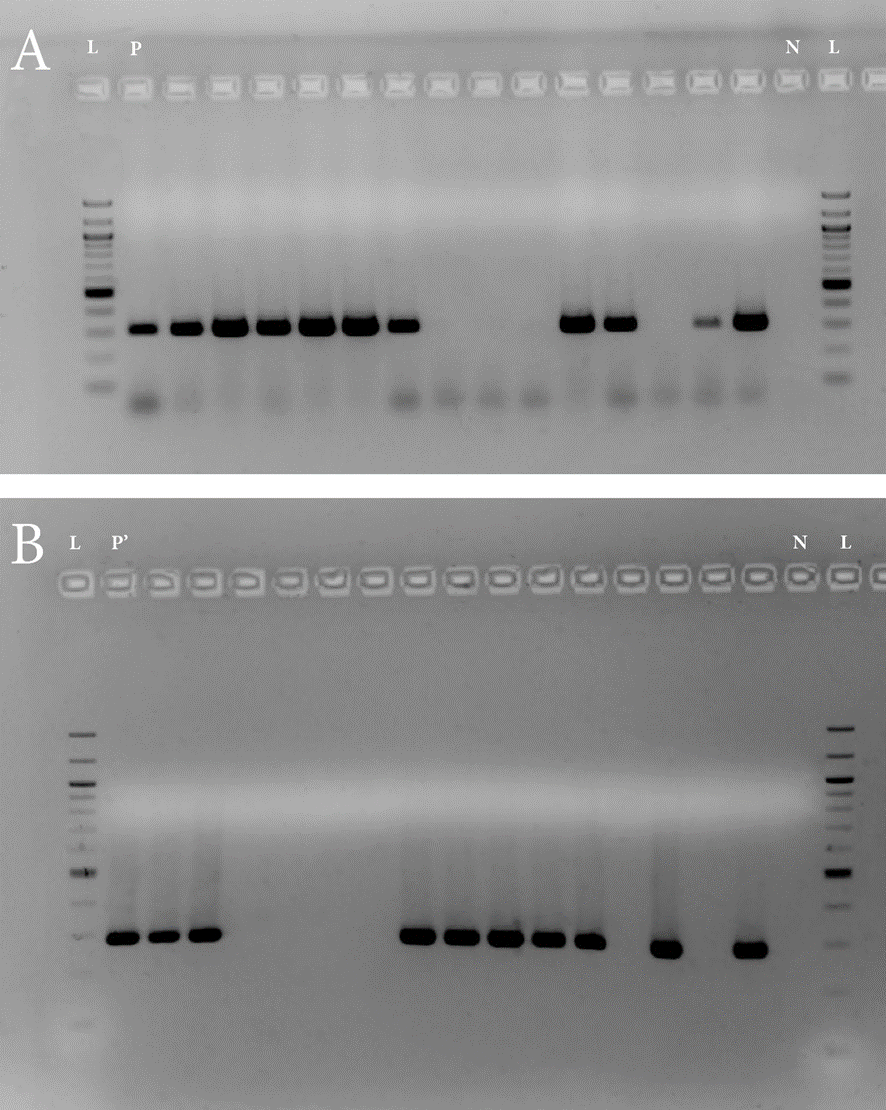


Supplementary figure 2. Electrophoretic analysis of RT-PCR products for detection of PPV (A) and HSVd (B) in established shoots 12 weeks from meristem rescue. L: 100 bp ladder; P: PPV positive control; P’: HSVd positive control; N: Negative control for water.
